# Investigations of the Sulfonated Poly(ether ether ketone) Membranes with Various Degrees of Sulfonation by Considering Durability for the Proton Exchange Membrane Fuel Cell (PEMFC) Applications

**DOI:** 10.3390/polym17162181

**Published:** 2025-08-09

**Authors:** Yinfeng Song, Zhenshuo Guo, Jiayi Yin, Mengjie Liu, Ivan Tolj, Sergey A. Grigoriev, Mingming Ge, Chuanyu Sun

**Affiliations:** 1 School of Electrical Engineering and Automation, Harbin Institute of Technology, Harbin 150001, China; 2 Guangdong Provincial Key Laboratory IRADS and DLS, Beijing Normal-Hong Kong Baptist University, Zhuhai 519087, China; 3 School of Engineering, Westlake University, Hangzhou 310030, China; 4 Shenzhen Key Laboratory of Polymer Science and Technology, College of Materials Science and Engineering, Shenzhen University, Shenzhen 518055, China; 5 Guangdong Provincial Key Laboratory of New Energy Materials Service Safety, College of Materials Science and Engineering, Shenzhen University, Shenzhen 518055, China; 6 College of Physics and Optoelectronic Engineering, Shenzhen University, Shenzhen 518060, China; 7 Suzhou Research Institute, Harbin Institute of Technology, Suzhou 215104, China; 8 Faculty of Electrical Engineering, Mechanical Engineering and Naval Architecture, University of Split, R Boskovica 32, 21000 Split, Croatia; 9 National Research University “Moscow Power Engineering Institute”, 14, Krasnokazarmennaya St., Moscow 111250, Russia; 10 National Research Centre “Kurchatov Institute”, 1, Akademika Kurchatova Sq., Moscow 123182, Russia; 11 HySA Infrastructure Center of Competence, Faculty of Engineering, North-West University, Potchefstroom 2531, South Africa; 12 A.N. Nesmeyanov Institute of Organoelement Compounds of the Russian Academy of Sciences, 28-1, Vavilova St., Moscow 119334, Russia

**Keywords:** degree of sulfonation, SPEEK membrane, proton exchange membrane fuel cell (PEMFC), Fenton test, chemical stability, proton conductivity, membrane electrode assembly, hydrogen energy system, power density, durability

## Abstract

The optimum degree of sulfonation (DS) for sulfonated poly(ether ether ketone) (SPEEK) membranes is determined by comprehensive characterization results, including proton conductivity, swelling ratio, water uptake, chemical stability, thermal stability, mechanical indicators, and proton exchange membrane fuel cell (PEMFC) performance. The PEMFC with a membrane electrode assembly containing a SPEEK-62 (DS = 62%) membrane realizes the power density of 482.08 mW/cm^2^, surpassing that of commercial Nafion-212 under identical conditions. In the crucial Fenton test for durability, the SPEEK-51 membrane demonstrated outstanding dimensional and chemical stability, with a decomposition time of up to 137 min, far surpassing the durability of SPEEK-62 or other membranes with a higher DS. The results indicate that in comparison to the SPEEK-67 membrane as reported in the literature, SPEEK membranes with a DS = 51~62% hold great potential for future applications in PEMFC, and further modifications of these membranes can be a promising approach to enhance the conductivity while maintaining good chemical stability.

## 1. Introduction

Polymer electrolyte membrane fuel cells (PEMFCs) are the most prospective electrochemical energy generation devices at present for transport and stationary applications as a result of their transient response time, industrial adaptability, extraordinary energy density, and environmental friendliness [1,2,3]. The current demand for the capacity growth of electric vehicles and the modernization of novel power systems have promoted the demand for high durability and low-cost PEMFC [4,5,6]. The membrane electrode assemblies (MEAs) act as the pivotal component in PEMFCs, and they are where the electrochemical reactions take place. It is important to prepare high-performance MEA which demonstrates excellent mass transfer in various components. The MEA contains three primary components: proton exchange membrane (PEM), gas diffusion layers (GDLs), and catalyst layers (CLs) [7,8,9]. The oxidation reaction of hydrogen takes place on the anode, where electrons (e^−^) and protons (H^+^) are formed under the action of the anode catalyst. The H^+^ formed on the anode passed through the PEM to the cathode, and the simultaneously formed free e^−^ are transported to the cathode through the external circuit to achieve electrical neutrality. While on the cathode, oxygen acts in the role of an oxidant, which reacts with the above-mentioned electrons and protons to generate water and release heat. Hence, water, heat, and the electric power are generated within the PEMFC concurrently. Furthermore, the water and thermal management is of great significance during the long-term PEMFC operation [10,11,12,13,14,15].

The PEM as the core material in PEMFCs is primarily employed to transport H^+^ and separate the gas reactants between the cathode and anode, which prominently affect the PEMFC output performance. At present, the most widely adopted commercial membrane is the perfluorosulfonic acid (PFSA) membrane. For instance, Nafion invented by DuPont (USA) in the 1960s possesses excellent proton conductivity, high chemical stability, and exceptional mechanical/thermal properties [16,17,18]. Although considerable modifications of pristine Nafion membranes have been carried out and the PEMFCs assembled with these membranes have demonstrated better output performance, the high cost and poor performance under elevated temperatures (above 80 °C) of Nafion impedes the commercial application of PEMFCs, such as the price of Nafion-212 membrane ranging from USD 500 to USD 1000 per square meter [19]. Meanwhile, the chemical, electrochemical, thermal, and mechanical degradation of the PEM in the PEMFC is a prominent obstacle for its commercialization in wide-scale applications [20]. During the PEMFC operation, mechanical stress may lead to lifespan reduction as a result of the existence of pinholes, perforations, cracks, and tears in the PEMs. Moreover, the operation of the PEMFC at various relative humidity (RH) conditions results in dimensional variations, which may affect the mechanical stability as well [21,22,23,24]. The employment of the PEMs at elevated temperatures accelerates the thermal degradation process. From the aspect of chemical degradation, the formed free radicals during the PEMFC operation attack the main chain of the PEM, which results in the decomposition of polymers, deteriorates the integrity of the PEM, and diminishes the durability and performance of the PEMFC. In practice, the PEM failure is caused due to the integrated influences of thermal, chemical, and mechanical degradation [25,26,27]. Therefore, enormous investigations have contributed to designing alternative PEMs and developing more cost-effective PEMs that possess the capability of operating at elevated temperatures, together with superior chemical, mechanical, and thermal stability [28,29].

As an outstanding representative of non-fluorine PEM, sulfonated poly(ether ether ketone) (SPEEK) is regarded as the most prospective vicarious PEM, which possesses regulable proton conductivity and outstanding mechanical/thermal stability. Previous investigations have concluded that a degree of sulfonation (DS) = 67% is the optimal DS for SPEEK membranes in PEMFC applications, and substantial investigations of SPEEK polymer membranes and their modifications have been reported, which mainly concentrated on medium or higher DS values (>67%) [30,31]. The modifications contain the synthesis of organic–inorganic hybrid PEMs by introducing nanoparticles, blending with organic components to form composite membranes, and employing various ionic liquids or crosslinking agents to enhance the comprehensive features of the pristine SPEEK membranes [32,33,34,35,36,37,38]. Even though the SPEEK membranes with high DS values may provide satisfactory proton conductivity, which is even close to Nafion, they also significantly impair the chemical stability, dimensional stability, and mechanical strength. As reported in the literature, SPEEK membranes with a DS that is too high exhibit excessive swelling behavior and even dissolve in aqueous solutions at high temperatures; so it is difficult to find commercial SPEEK membranes in the market [39,40,41]. Nevertheless, the properties of SPEEK membranes which possess a lower DS remain largely unexplored and warrant further investigations. The chemical stability and proton conductivity of SPEEK membranes are highly correlated with the DS, and it is possible to adjust the DS value through altering the reaction conditions and processing factors, including reaction time and sulfonation temperature [42,43,44]. The above-mentioned drawbacks may in turn bring about a prominent reduction in the lifespan of the PEMFC. To elucidate the effect of DS on different characteristics of the pristine SPEEK membranes for long-durable PEMFC applications and to obtain the SPEEK membrane with excellent chemical stability, attempting to utilize the SPEEK membrane with a low DS may overcome the defects. These drawbacks are prone to chemical degradation and severe swelling behaviors.

In this work, a series of SPEEK membranes with various DS values were prepared and investigated, including comprehensive stability characterization (mechanical, chemical, and thermal) and other physicochemical features to assess their potential applications for PEMFCs. Moreover, the actual single-cell performance of MEAs made from SPEEK membranes with different DS values in PEMFCs was conducted in order to demonstrate the optimal DS value range of SPEEK membranes for long-term stable and high-performance PEMFC operation.

## 2. Materials and Methods

### 2.1. Materials

PEEK grade 450P powders were acquired from Victrex Company (Lancashire, UK). Concentrated sulfuric acid (H_2_SO_4_, 95–98 wt%) was purchased from Xilong Scientific Company (Guangdong, China). N,N-dimethylformamide (DMF) was procured from Tianjin Fuyu Chemical Company (Tianjin, China). PFSA membranes (Nafion-212) were acquired from Sinero Technology Company (Suzhou, China), which were latter immersed in 3 wt% hydrogen peroxide solution, 1 mol/L sulfuric acid, and bidistilled water under 80 °C before use, as typically reported and pretreated in the literature [45]. All SPEEK membranes were soaked in 1 mol/L dilute sulfuric acid solution for the whole day before use [46]. All chemical reagents belong to the analytical grade and were employed without further purification.

### 2.2. Sulfonation of Poly (Ether Ether Ketone)

Initially, 2 g of pre-dried PEEK powders was slowly incorporated in 20 mL of concentrated H_2_SO_4_ under continuous mechanical stirring, maintained at ambient temperature until complete dissolution. The homogeneous solution was then subjected to sulfonation at 55 °C in a water bath with vigorous agitation for predetermined durations ranging from 1.5 to 4 h to synthesize SPEEK polymers with tunable DS, as shown in Table 1. To terminate the reaction, the viscous solution was poured into an ice-water bath and precipitated, and water was constantly replaced for washing until the washing liquid was neutral (pH = 6–7). The formed SPEEK fibers after washing were dried in an oven at 20 °C for 12 h and then under 80 °C for 24 h.

### 2.3. Synthesis of SPEEK Membranes

SPEEK fibers with different DS values were dissolved in DMF under magnetic stirring at 60 °C for 30 min to achieve homogeneous polymer solutions. As reported in the literature, DMF is selected as the optimal solvent compared to DMAc, NMP, and DMSO [46,47,48,49]. These solutions subsequently underwent vigorous sonication with an ultrasonic homogenizer (QQ6-200A, Shanghai Qiqian Electronic Technology Co., Ltd., Shanghai, China) for 30 min to ensure molecular-level dispersion. The obtained solutions were poured into the glass Petri dish and maintained for 12 h under 60 °C, followed by further heating for 24 h under 80 °C to form SPEEK membranes with a thickness of 55 ± 10 μm. The SPEEK membranes were soaked in 1 mol/L H_2_SO_4_ solution to activate for the whole day and labeled as SPEEK-X (X = 34, 43, 51, 62, 72), where X indicates DS.

### 2.4. Characterizations and Measurements

#### 2.4.1. Morphology Analysis

The surface morphologies of the PEMs were directly examined by employing a scanning electron microscope (SEM, SU8010, Hitachi, Japan) with 10 kV as operating voltage. Firstly, conductive adhesive was adopted to stick a tiny slice of PEM on the surface of the sample stage. Secondly, the sample was sprayed with gold to form the gold coating on the sample surface. Finally, the prepared sample was placed in the SEM instrument for observation.

#### 2.4.2. Chemical Structure Analysis

Fourier transform infrared (FTIR) spectroscopy (Great 20, Zhongke Ruijie Technology Company, Tianjin, China) was utilized to analyze the membrane structure. All samples should be dried at 60 °C for 12 h before testing, and then cut into small square pieces of 2 cm × 2 cm to conduct the test. The measurements were recorded over a wavenumber range between 500 and 4000 cm^−1^, and the resolution was 1 cm^−1^.

#### 2.4.3. Mechanical Properties

The mechanical property measurement was carried out by the tensile testing machine (UTM2202, SUNS Instrument Co., Ltd., Shenzhen, China), and the specimen was cut to form a rectangular configuration. The specimen was 1.6 cm in width and 5 cm in length, and the test was carried out with the stretching rate of 5 mm/min.

#### 2.4.4. Swelling Ratio and Water Uptake

The swelling ratio (SR) and water uptake (WU) of the SPEEK series membranes were evaluated by using a circular membrane piece with a diameter of 2 cm. Initially, the piece was pre-dried at 60 °C for 12 h, and its dry weight (W_d_) and dry thickness (t_d_) were measured. The piece was then soaked in bidistilled water for 12 h at room temperatures, after which the wet weight (W_w_) and wet thickness (t_w_) were recorded. The SR and WU were subsequently calculated through Equations (1) and (2).

SR = (t_w_ − t_d_)/t_d_ × 100% (1)

WU = (W_w_ − W_d_)/W_d_ × 100% (2)

#### 2.4.5. Thermal Stability

The thermal degradation process for the membrane was estimated by employing thermogravimetric analysis (TGA, DZ-TGA201, Nanjing Dazhan Testing Instrument Co., Ltd., Nanjing, China). Firstly, the specimen was placed in the TGA testing instrument and dried for 2 h under 80 °C to fully expel any moisture from the sample. Then, the dried sample mass was input into the testing instrument, and the testing process was initiated. In these tests, the temperature rose from 24 °C to 700 °C at the ramp rate of 10 °C/min under the air atmosphere.

#### 2.4.6. Degree of Sulfonation and Ion Exchange Capacity

Acid–base titration was employed to evaluate the IEC of the PEM. Initially, the membrane was pre-dried for 12 h under 60 °C to measure its dry weight (W_0_). Afterwards, the PEM was immerged in a 3 mol/L NaCl solution for the whole day, enabling the sufficient exchange of protons with sodium ions. Finally, the obtained solution was titrated with the 0.02 mol/L NaOH solution (C_NaOH_), and the IEC was determined based on the reacted volume of NaOH (V_NaOH_); the calculation process is described in Equation (3). Moreover, the DS of SPEEK was determined by Equation (4) based on the IEC values [30].

IEC = V_NaOH_ × C_NaOH_/W_0_ (3)

DS = 288 × IEC/(1000 − 80 × IEC)(4)

#### 2.4.7. Proton Conductivity Measurement

**Figure 1:**
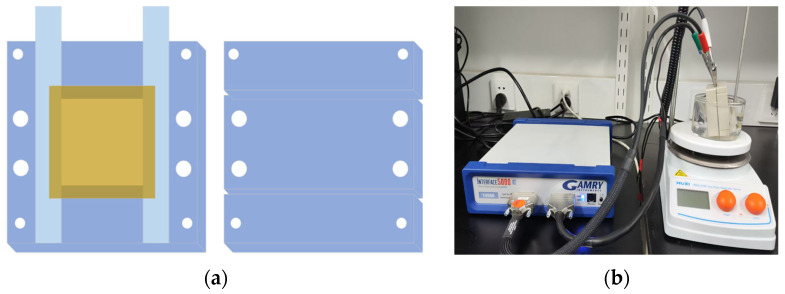
(**a**) Structural diagrammatic sketch of PEM conductivity testing fixture. (**b**) Electrochemical impedance spectroscopy (EIS) measurement system of membrane.

Proton conductivity (σ) was evaluated in bidistilled water in the temperature of 24, 30, 40, 50, 60, 70, and 80 °C using a two-electrode impedance spectrometer (Gamry Instruments Interface 5000E^TM^, Gamry Electrochemical Instrument Company, Warminster, PA, USA, operating at 1–1000 kHz). A membrane sample (3 cm × 3 cm) was sandwiched between two platinum wires and secured with Teflon blocks, as shown in Figure 1a; it was designed and kindly provided by Wuhan Chuxin Technology Co., Ltd (Wuhan, China). Before the measurement, the PEM was equilibrated in bidistilled water for 10 min at the target temperature, after which its impedance (R, Ω) was recorded, as shown in Figure 1b. σ (mS/cm) can be then calculated according to Equation (5):

σ = L/(A × R)(5)

where A (cm^2^) is the cross-sectional area of the sample, and L (cm) is the distance between two electrodes.

#### 2.4.8. Oxidative Stability Measurement

The oxidative stability of the PEM was assessed by adopting the reagent of Fenton (3 wt% H_2_O_2_ with 18 ppm FeSO_4_). Rectangular PEM samples (2 cm × 2 cm) were dried for 12 h under 70 °C to determine the dry weight (W_0_). Each specimen was then immersed in the Fenton solution heated in a 60 °C water bath, and mass (W_1_) was recorded. The mass change (W_c_) is calculated using Equation (6), while the onset times of visible crack formation and complete mechanical disintegration were recorded.

W_c_ = (W_1_ − W_0_)/W_0_ × 100% (6)

#### 2.4.9. Fabrication of Membrane Electrode Assemblies (MEAs) and Evaluation of PEMFC Single-Cell Performance and Open Circuit Voltage Durability Test

**Figure 2:**
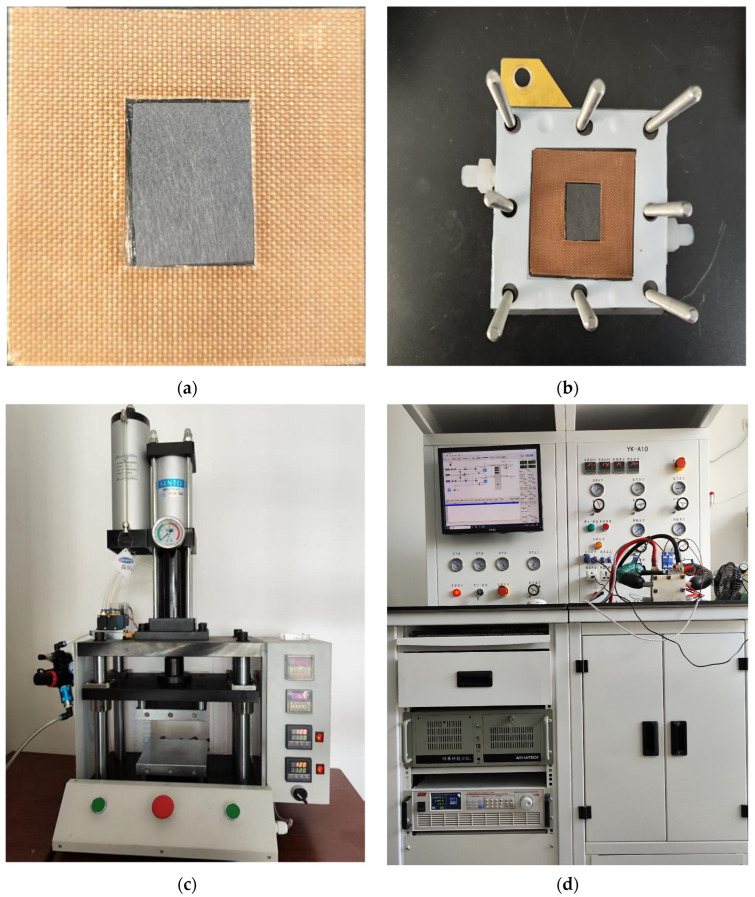
(**a**) MEAs after hot press at 120 °C under 3 MPa for 5 min; (**b**) single cell with MEAs; (**c**) hot press equipment; (**d**) PEMFC single-cell test system.

The hot pressing technique was employed for the MEA fabrication, which contains the PEM with a productized gas diffusion electrode (GDE, with cathode Pt loading of 0.3 mg/cm^2^ and anode Pt loading of 0.1 mg/cm^2^, Suzhou Sinero Technology Co., Ltd., Suzhou, China) on both sides at 120 °C under 3 MPa for 5 min, as demonstrated in Figure 2a,c. The performance of the PEMFC single cells assembled with prepared MEAs were measured by employing the PEMFC single-cell test system (YK-A10-100W, Dalian Yuke Innovation Technology Co., Ltd., Dalian, China) with the active area of 5 cm^2^, as shown in Figure 2b,d. All the single cells were assembled using a torque wrench set to 6 N·m. Before testing, the back pressure on both the cathode and anode was gradually increased to 0.1 MPa. The polarization and power density curves of the PEMFC single cells containing various MEAs were measured under 70, 75, and 80 °C with 100% relative humidity (RH). Both sides of the anode and cathode were supplied with hydrogen and air, respectively, and the anode and cathode stoichiometries values were selected as 1.5 and 2.5, respectively. Prior to testing, the PEMFC single cell was activated under a fixed potential of 0.2 V until a stable current was achieved. In constant current mode, the current was increased by 0.1 A every two minutes, ranging from 0 A to the predetermined maximum current. The current–voltage polarization curves of the PEMFC single cells assembled with all membranes were then recorded. The open circuit voltage (OCV) durability test of all the membranes was carried out at 70 °C and 50% RH. The gas flow rate and back pressure of the cathode and anode were consistent with the working conditions of the single-cell performance test. The stoichiometric ratios of the cathode and anode were 1.5 and 2.5, and the back pressure was 0.1 MPa.

## 3. Results and Discussion

### 3.1. FTIR Spectroscopy

**Figure 3:**
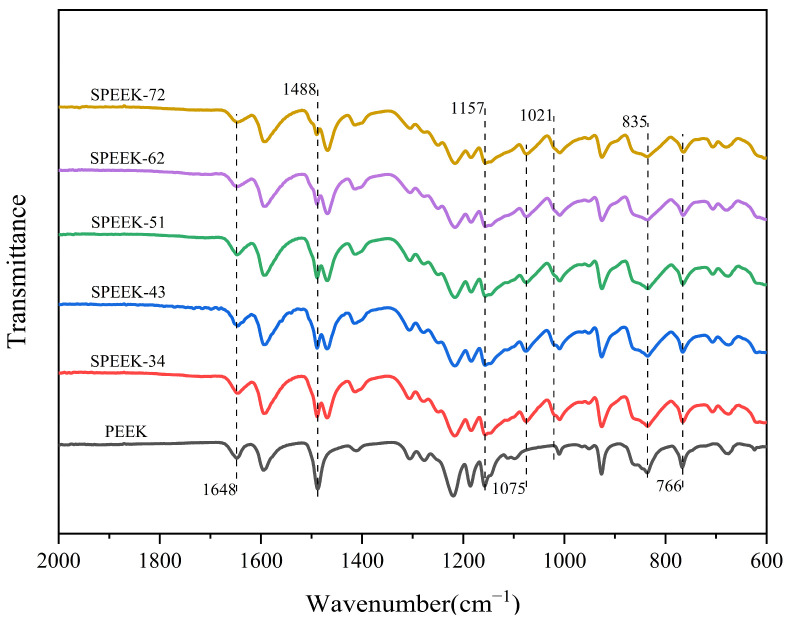
The FTIR spectrum of SPEEK membranes with different DS values and pristine PEEK powder.

FTIR spectroscopy was adopted for determining the presence of sulfonic acid groups and other chemical bonds in the obtained SPEEK membranes. The expected analogous FTIR spectra are demonstrated in Figure 3 for the SPEEK membranes. It can be observed that the peak at approximately 1488 cm^−1^ corresponds to C–C skeletal vibration bands [31]. The out-of-plane bending vibration peaks at 835 cm^−1^ and the C-H absorption peaks at 1157 cm^−1^ for the SPEEK series membranes were obviously weakened compared to pristine PEEK powder, which is due to the incorporation of -SO_3_H groups. Moreover, the bands at 1648 cm^−1^ are assigned to the C=O stretching vibration. Due to the sulfonation process which introduces -SO_3_H groups and substitutes hydrogen atoms of the benzene ring, the characteristic peaks of -SO_3_H groups are rather obvious: with the symmetric stretching vibration peaks of O=S=O bonds at 1075 cm^−1^, the stretching absorption peaks of O=S=O bonds at 1021 cm^−1^, and the symmetric stretching vibration peaks of S-O bonds at 766 cm^−1^ [50]. Apparently, the incorporation of the -SO_3_H groups into the benzene ring of the PEEK backbone structure and the peak intensity accordingly enhanced with the DS increase as well, revealing the consistency between the chemical structure of SPEEK series PEMs and FTIR characterization results.

### 3.2. SEM

**Figure 4:**
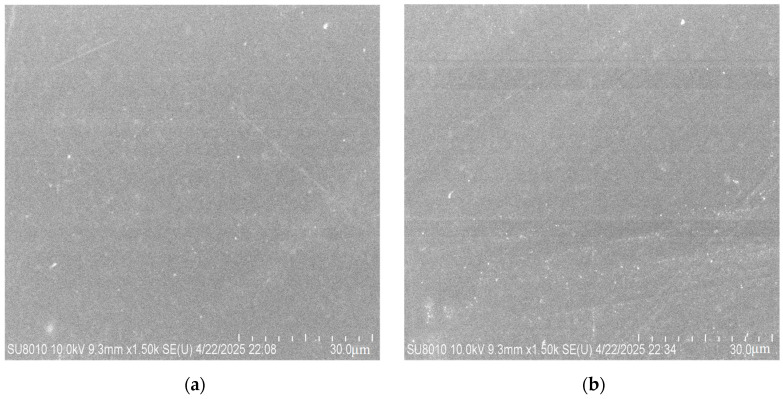

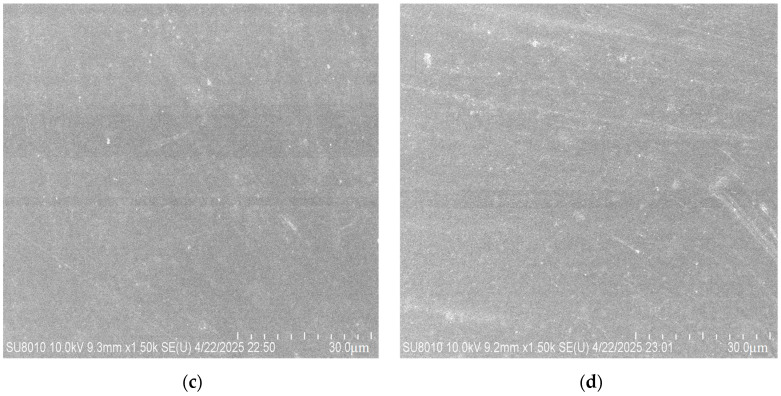
The top view morphology of various SPEEK PEMs: (**a**) SPEEK-43, (**b**) SPEEK-51, (**c**) SPEEK-62, (**d**) SPEEK-72.

To investigate the morphology of the SPEEK membranes, surface area profiles were characterized by SEM. Figure 4 reports the top view morphology of SPEEK PEMs with various DS values, respectively. The outcomes demonstrate dense and homogeneous morphology in terms of all the SPEEK membranes.

### 3.3. Physical Properties

Moisture content critically governs proton conduction in PEMs by mediating dual transport mechanisms: (i) the vehicular mechanism (H_3_O^+^ migration through broader water channels) and (ii) the Grotthuss mechanism (proton hopping via hydrogen-bond networks). The retained moisture occupies the unbound spatial voids within polymeric matrices, thereby inducing structural expansion. While these water-dependent processes enhance conductivity, excessive hydration induces pronounced membrane swelling that compromises mechanical integrity and operational durability. As quantified in Table 2, both WU and SR exhibit progressive enhancement with the increasing DS, while the mechanical properties are exactly the opposite. As the DS increases, the polymer backbone becomes more hydrophilic, leading to an increase in water uptake and plasticization of the material. This results in a reduction in the mechanical strength of the membrane. The water molecules weaken intermolecular forces between polymer chains, thus reducing the tensile strength and elongation at break. This process is called “polymer softening”. While the increase in the DS can lead to a higher degree of chain mobility due to the introduction of polar groups, these groups disrupt the rigidity of the polymer, making it more flexible but less resistant to external stresses.

Specifically, SPEEK membranes demonstrate a WU escalation from 7.3 to 43.3% and an SR increase from 4.2 to 15.4% when sulfonation duration extends from 1.5 to 4 h at 55 °C. As reported in the literature, the SR and WU have a remarkable influence on the proton conductivity and mechanical properties of SPEEK membranes [51].

### 3.4. TGA

**Figure 5:**
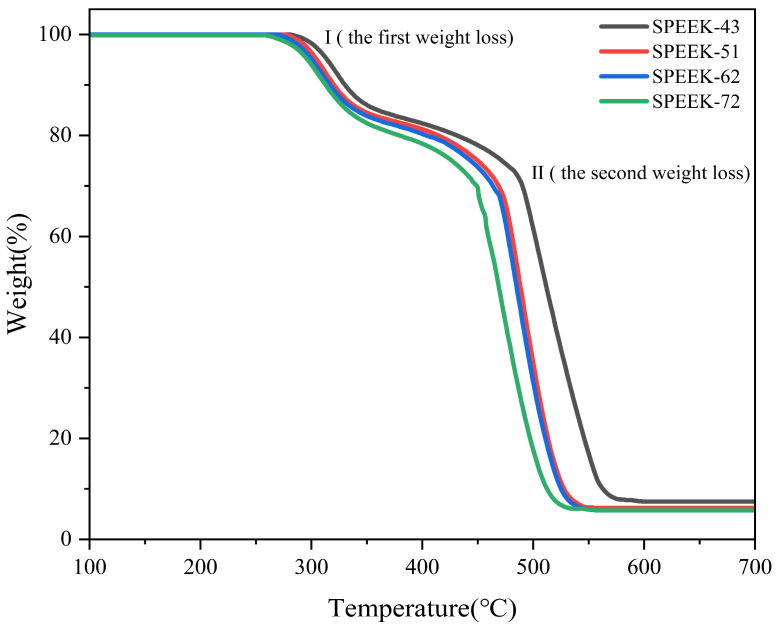
The TG curves of SPEEK-43, SPEEK-51, SPEEK-62, and SPEEK-72.

The thermal stability of SPEEK PEMs with multiple DS values was measured and determined by TGA, as shown in Figure 5. All the membranes show similar trends in terms of the TG curves and demonstrate two thermal decomposition sections, labeled I-II. The first weight loss (labelled I) is revealed at ca. 300 °C and is attributed to the decomposition of the -SO_3_H groups in the SPEEK backbone. The second thermal decomposition (labelled II) is at ca. 500 °C, and this can be attributed to the decomposition of the benzene rings of the SPEEK membranes. The investigations suggest that the SPEEK-72 sample has the lowest onset temperature for weight loss compared to the other SPEEK membranes. Hence, the incorporation of a larger quantity of -SO_3_H groups into the SPEEK polymer main chain results in a decrease in the thermal stability. The phenomenon can be explained as follows: as the DS increases, the polymer becomes more hydrophilic. This change in polarity causes the polymer to absorb more water, which acts as a plasticizer. The increase in chain mobility lowers the glass transition temperature Tg, making the material more susceptible to deformation under heat. Hence, -SO_3_H groups can lose their acidic protons at elevated temperatures, which can lead to the formation of sulfur trioxide (SO_3_) or other reactive intermediates. These species can attack the polymer backbone or neighboring sulfonic acid groups, leading to further degradation and loss of thermal stability. At elevated temperatures (≥80 °C), the SPEEK membranes with a low or moderate DS possess higher stability during long-term operation and application.

### 3.5. IEC

The IEC reflects the most critical features of SPEEK membranes. The influence of various sulfonation reaction durations on the IEC is reported in Table 2. As the sulfonation reaction time lasts, more -SO_3_H groups are incorporated into the backbone of the PEM structure. The hydrophilicity of the -SO_3_H groups enhances both the Grotthuss and vehicle mechanisms of proton conduction in the SPEEK membrane, ultimately promoting the IEC. The obtained DS results based on the IEC of various SPEEK membranes are also reported in Table 2.

### 3.6. Proton Conductivity

**Figure 6:**
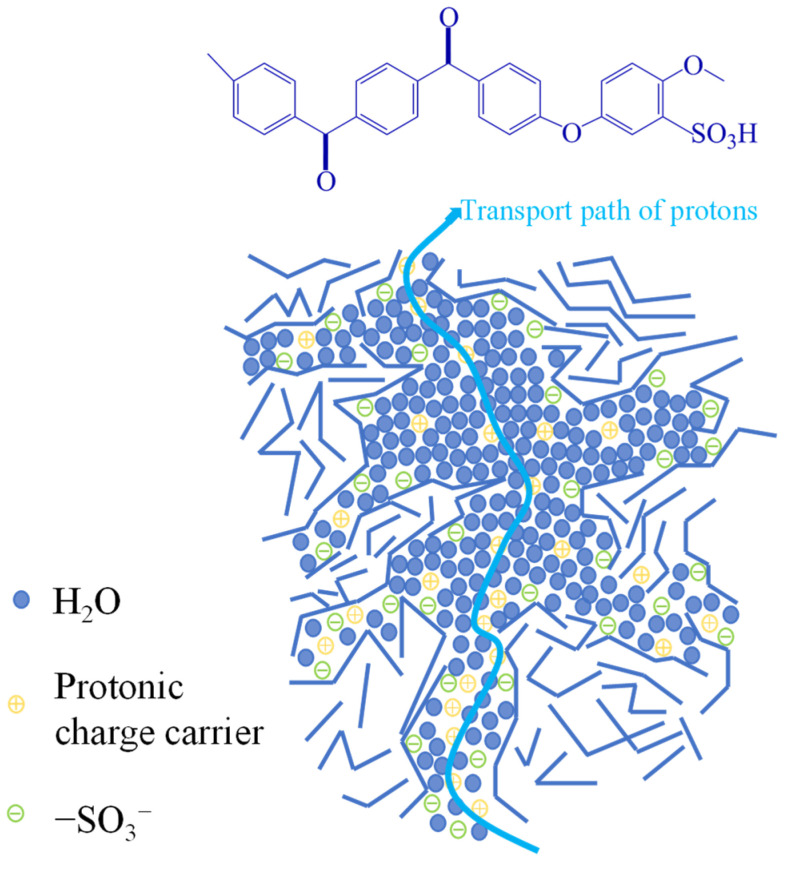
Proton transfer mechanism of SPEEK membrane.

For PEMs, the quantity of functional -SO_3_H groups and the corresponding WU at different temperatures have remarkable influences on the proton conductivity. With increasing DS, additional -SO_3_H groups are incorporated into the polymer, enhancing hydrophilicity and resulting in the adsorption of more moisture through the formation of hydrogen bonds. Moreover, this accelerates the vehicle mechanism by providing successive hydrophilic pathways. Therefore, a higher DS enhances the proton conduction capability of SPEEK membranes by promoting the quantity of protonated active sites (-SO_3_H groups) and enabling the formation of water-regulated channels for H^+^. The proton transfer mechanism of the SPEEK membrane is shown in Figure 6.

**Figure 7:**
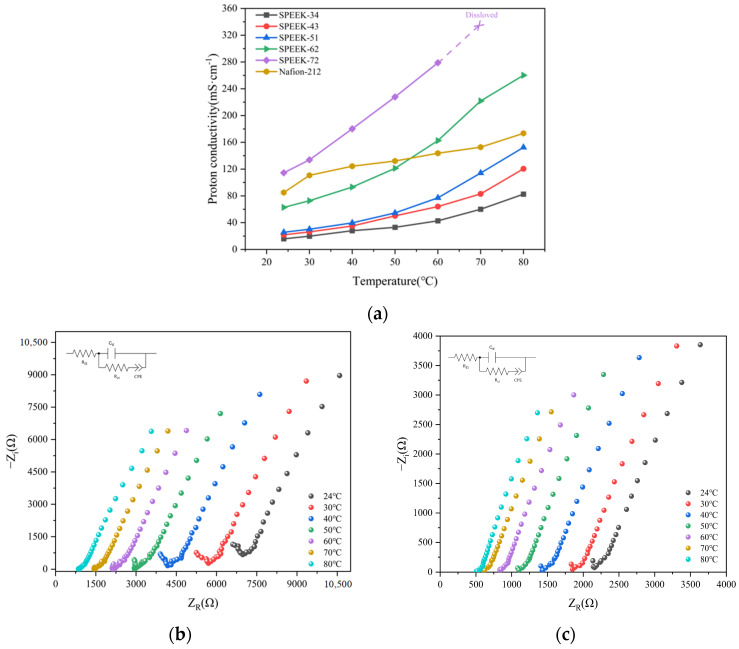
(**a**) Proton conductivity of SPEEK-34, SPEEK-43, SPEEK-51, SPEEK-62, SPEEK-72, and Nafion-212 membranes; (**b**) Nyquist diagram of SPEEK-51 at different temperatures (24–80 °C); (**c**) Nyquist diagram of SPEEK-62 at different temperatures (24–80 °C).

Obviously, owing to the smaller number of -SO_3_H groups in the SPEEK-34 membrane, the proton conductivity data obtained across the entire tested temperature range is far below the SPEEK membrane with higher DS values. Additionally, the solubility of SPEEK-34 fibers in DMF solvent is very poor, which creates challenges in the membrane preparation process. Due to its extremely low proton conductivity (<60 mS/cm), SPEEK-34 is not considered suitable for PEMFC applications. Therefore, only its proton conductivity and FTIR spectra were characterized to provide basic reference data, and the SPEEK-34 membrane is not further discussed in subsequent sections. Moreover, the SPEEK-72 membrane swelled severely in a water bath at 70 and 80 °C, leading to rupture and making it impossible to obtain their proton conductivity at those temperatures. The temperature influence on the proton conduction capability for the SPEEK membranes with various DS values was conducted, and the outcomes are reported in Figure 7a. It indicates that as temperature rises, the proton conductivity is enhanced because higher thermal energy enables protons to overcome fixed charges within the PEM. When the PEM absorbs water, the -SO_3_H groups interact with H_2_O molecules, causing the hydrophilic domains to swell and supply more pathways for H^+^ conduction via the membrane. The σ of the SPEEK-51 and SPEEK-62 membranes was evaluated by employing electrochemical impedance spectroscopy (EIS) at multiple temperatures, as presented in Figure 7b,c. Based on the Nyquist plot obtained from the EIS results, the resistance is determined by applying the equivalent circuit, and the σ of the SPEEK-51 membrane shows a clear upward trend over the temperature range of 24–80 °C.

### 3.7. Oxidative Stability

Among the various degradation courses, chemical degradation is critical for the exploration of the above-mentioned PEMs. Sulfonation introduces highly polar functional groups into the polymer, which can make the material more susceptible to hydrolysis and oxidation. The hydrolysis indicates that the -SO_3_H groups are hydrophilic and attract water, leading to an increase in water uptake. In highly sulfonated PEEK, the -SO_3_H groups may dissociate into their protonated form (H^+^) in the presence of water. The excess protons can attack the polymer backbone, leading to hydrolytic degradation of the ether and ketone linkages in the PEEK structure. This breakdown of the polymer backbone compromises its chemical stability.

During the PEMFC operation, fragmentary oxygen reduction reactions occur at the cathode, and oxygen/hydrogen can penetrate across the PEM from the cathode/anode to the other one, which results in the generation of free radicals. Peroxide radicals, which may bring about chemical degradation of the PEM, play a central role in this process. The Fenton reagent is commonly used in the accelerated stressed test, which can be utilized for chemical stability evaluation of PEMs to simulate the real-time chemical degradation. In the Fenton test, certain metallic species, including Fe^2+^, were employed for the decomposition of H_2_O_2_ to create free radicals, as presented in Equations (7) and (8), and the generated free radicals may attack the PEM and degrade the backbone of SPEEK membranes [52,53,54].

H_2_O_2_ + Fe^2+^ → Fe^3+^ + HO· + OH^−^ (7)

Fe^3+^ + H_2_O_2_ → Fe^2+^ + HOO· + H^+^ (8)

**Figure 8:**
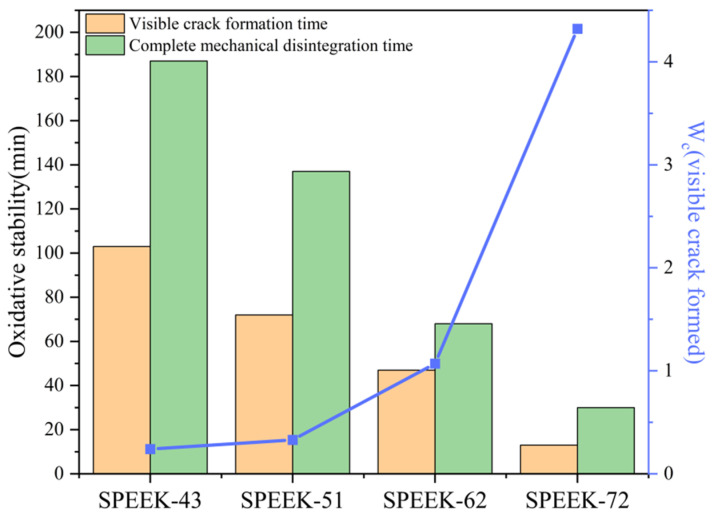
Visible crack formation time, complete mechanical disintegration time, and W_C_ when visible crack formed for SPEEK membranes with various DS.

Figure 8 shows the visible crack formation and the complete mechanical disintegration time of SPEEK membranes with different DS values during the Fenton test, along with the recorded W_c_ at the point when visible cracks appear, as shown by the blue line in the figure. It can be noted that SPEEK membranes with a lower DS exhibit higher stability compared to the higher ones. Specifically, the complete degradation time of the SPEEK membrane with the DS = 51% is twice as long as that of the membrane with a DS = 62%. When visible cracks form, the WU rate of the SPEEK membrane with a DS = 62% reaches an astonishing 107%, whereas the WU rate of the membrane with a DS = 51% remains consistently below 35%. Therefore, the oxidation stability of the SPEEK membrane with a DS = 51% far exceeds that of the membrane with a DS = 62%.

### 3.8. PEMFC Single-Cell Performance

**Figure 9:**
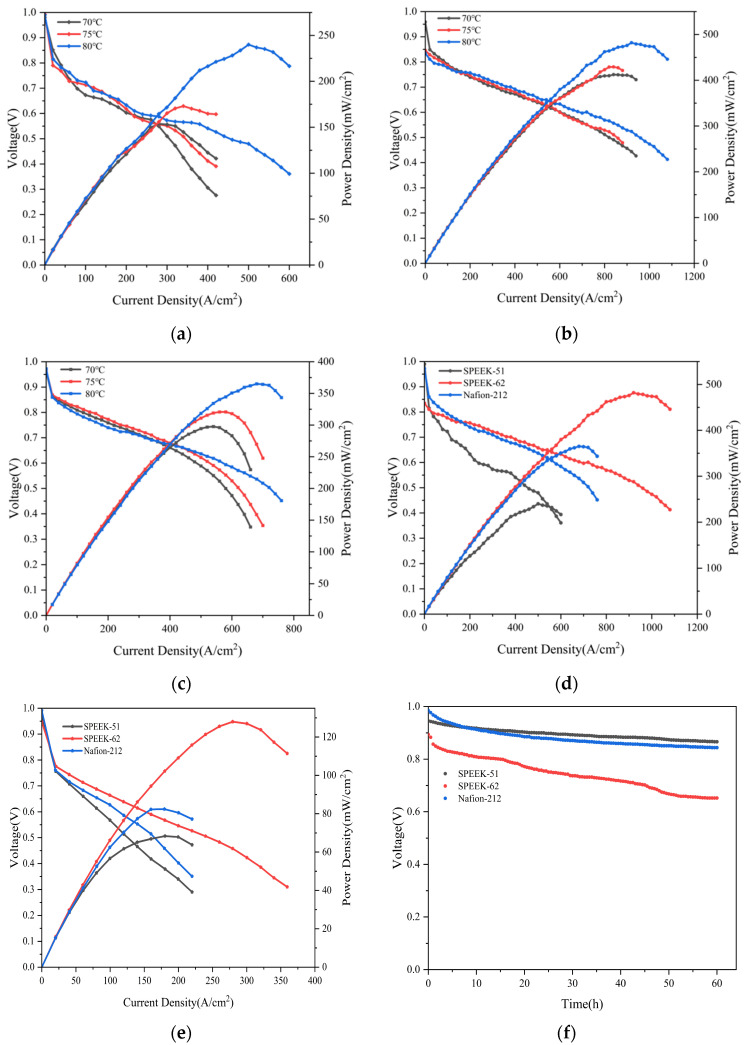
PEMFC performance of MEAs prepared by (**a**) SPEEK-51 membrane; (**b**) SPEEK-62 membrane; (**c**) Nafion-212 membrane at various temperatures; (**d**) PEMFC performance of MEAs assembled with different PEMs under 80 °C and under 100% RH condition; (**e**) PEMFC performance of MEAs assembled with different PEMs under 70 °C and 50% RH condition; (**f**) OCV curve of the SPEEK-51, SPEEK-62, and Nafion-212 under 80 °C and 100% RH.

Figure 9a–c display the current density–voltage polarization curves of the PEMFC single cells containing SPEEK-51, SPEEK-62, and Nafion-212 membranes at the temperatures of 70, 75, and 80 °C under 100% RH. Based on the PEMFC single-cell outcomes, when the operation temperature rises to 80 °C, the peak power densities of single cells with all MEAs containing SPEEK and Nafion membranes demonstrate a remarkable upward tendency. During the activation process, several MEAs containing SPEEK-72 membranes were damaged at 70 °C and 100% RH, resulting in severe attenuation of open circuit voltage and extremely unstable current–voltage polarization curves. Meanwhile, it could be found during the experiments that these MEAs exhibited extremely severe swelling behavior just after a short operation period, and cracks even appeared at the membrane edges. Consequently, the comprehensive characterizations revealed that the SPEEK-72 membrane is not appropriate for long-term application in PEMFCs. The power density of the PEMFC single cell containing the SPEEK-62 membrane at three testing temperatures significantly exceeded that of the Nafion-212 membrane. And its peak power density achieved 482.08 mW/cm^2^ under 80 °C, far exceeding the 365.16 mW/cm^2^ of Nafion-212 under the identical testing conditions. The measured peak power density of the Nafion-212 membrane is close to what is reported in the literature [55]. From Figure 9b, it can be seen that during the testing process at three consecutive temperature points, its open circuit voltage continuously declined from 0.959 to 0.835 V. In the Fenton test, the WU rate of the SPEEK-62 membrane reached 107% within just 47 min, and visible cracks were formed. Although the SPEEK-62 membrane possesses excellent PEMFC single-cell performance in PEMFCs, its rapid degradation under operating conditions renders it unsuitable for long-term application. By comparison, the PEMFC single cell containing the SPEEK-51 membrane achieved a peak power density of 236.93 mW/cm^2^ under 80 °C and 100% RH as the operating condition. While the output performance of the SPEEK-51 membrane is not as high as Nafion-212, the SPEEK-51 membrane exhibits exceptional stable performance during the entire testing process. Figure 9d comprehensively compares the PEMFC single-cell performance of MEAs containing SPEEK-51, SPEEK-62, and Nafion-212 membranes under the 80 °C and 100% RH condition.

From the perspective of the proton transport mechanism, water plays a vital role as a medium for proton transport, making it an indispensable component of PEMFC operation [56]. In single-cell performance testing, the water content in the MEA is completely controlled by the gas humidity. Especially for sulfonated polymers that rely on high IEC values, the effect of humidity on electrochemical performance is fatal [57]. Figure 9e records the peak power density curves of single cells based on SPEEK-51, SPEEK-62, and Nafion-212 membranes operating at 50% humidity. When the relative humidity of the test decreases from 100% to 50%, the peak power density of SPEEK-51 decreases from 153.78 mW/cm^2^ to 68.3716 mW/cm^2^, a decrease of about 55.5%, and the performance output loss is significantly lower than 69% and 72.3% of SPEEK-62 and Nafion-212. At the same time, the peak power density of the SPEEK-51 membrane at 50% relative humidity is very close to 80.4804 mW/cm^2^ of Nafion-212; so this result may be due to the fact that the SPEEK-51 membrane has a stronger ability to retain water molecules than Nafion. Given that humidity has a limited effect on the proton conduction of the SPEEK-51 membrane, the dehydration of the MEA containing the SPEEK-51 membrane is not as severe as that of the Nafion membrane at RH = 50%, and the increase in ohmic resistance caused by membrane drying is lower than that of the Nafion membrane. Thus, the performance decay of the PEMFC single cell equipped with the SPEEK-51 membrane is lower than that of the Nafion-212 membrane under the same conditions.

OCV durability experiments were conducted on PEMFCs assembled with SPEEK-51, SPEEK-62, and Nafion-212 to study the long-term stability of PEMs during PEMFC operation. The difference in the initial OCV between the PEMFC single cells containing SPEEK-51 and SPEEK-62 may be caused by the different hydrogen crossover rates. Figure 9f shows that under the operating conditions of 70 °C and 100% RH, the OCV of the single cells with SPEEK-51, SPEEK-62, and Nafion-212 decayed by 0.08 V, 0.242 V, and 0.141 V, respectively, with decay rates of 1.33, 4.03, and 2.35 mV h^−1^, respectively. The rapid OCV decay for PEMFC with SPEEK-62 is caused by the membrane absorbing too much water for a long time in a high-temperature and high-humidity environment. Firstly, water swelling reduces the concentration of sulfonic acid groups in the membrane, resulting in a decrease in proton conductivity. Secondly, excessive water uptake and dimensional expansion may also weaken the molecular interaction between the membrane and the catalyst layer, thereby increasing the interfacial resistance between the membrane and the catalyst layer, and even causing the catalyst layer to peel off from the membrane, further reducing the durability of the MEA [58]. In addition, when there is an excessive water hydrate level, the gaps between the molecular chains become larger, and hydrogen permeation increases, which also reduces the voltage of the PEMFC assembled with membranes [59]. Finally, the cathode side is immersed in water for a long time, so that oxygen cannot quickly reach the active sites of the catalyst, thereby slowing down the redox reaction and greatly reducing the performance of the membrane [60]. In comparison, the OCV degradation of SPEEK-51 is the smallest. It can be seen that the SPEEK-51 membrane does not suffer from serious hydrogen permeation and possesses high durability.

## 4. Conclusions

This work investigated the influence of the DS on the mechanical/thermal stability, WU behavior, IEC, proton conductivity, and chemical stability of SPEEK membranes for the PEMFC applications. The analysis and characterization outcomes indicated that the chemical, mechanical, and thermal stability of the SPEEK series PEMs declined with the increase in DS, whereas the proton conductivity demonstrated the opposite trend. Based on the relatively poor performance of the SPEEK-62 membrane in the Fenton test and PEMFC single-cell testing, it can be inferred that the SPEEK-62 membranes are not the optimal DS for PEMFCs in terms of long-time operation. These results indicate that when durability is taken into account, the conclusion can be different from the conventional conclusions reported by the literature, which typically reports that SPEEK membranes with DS = 67% are the most suitable for PEMFC applications. In contrast, the SPEEK-51 membrane exhibits extremely excellent mechanical, thermal, and chemical stability. In particular, in the Fenton accelerated aging test, SPEEK-51 exhibited superior chemical stability performance, equivalent to two times that of the SPEEK-62 membrane. Furthermore, the PEMFC single cell containing SPEEK-62 and SPEEK-51 membranes achieved a power density of 482.08 and 236.93 mW/cm^2^ under 80 °C and 100% RH during testing, respectively. These results suggest that the SPEEK membranes with DS ranging from 51 to 62% are more suitable for the PEMFC applications due to their favorable chemical stability, durability, and excellent PEMFC performance. This paper provides important guidance and reference for further optimizing the comprehensive performance of SPEEK membranes in the future and promoting the commercialization of SPEEK series membranes.

## Figures and Tables

**Table 1:** DS corresponding to different sulfonation times at 55 °C.

Sulfonation Time (h)	Temperature (°C)	DS (%)
1.5	55	34
2.3		43
3		51
3.5		62
4		72

**Table 2:** Thickness, WU, SR, IEC, tensile strength, and proton conductivity results of SPEEK membranes with multiple DS and Nafion-212.

Sample	Thickness (μm)	WU (%)	SR (%)	IEC (mmol/g)	Tensile Strength (MPa)	σ at 60 °C (mS/cm)
SPEEK-34	55	7.30	4.20	1.08	38.8	42.78
SPEEK-43	53	16.20	5.90	1.30	35.6	64.10
SPEEK-51	57	22.10	7.60	1.55	32.8	77.16
SPEEK-62	58	35.60	12.70	1.84	30.5	162.60
SPEEK-72	60	43.30	15.40	2.08	28.3	278.95
Nafion-212	51	15.70	10.20	0.97	32.4	143.73

## Data Availability

The data presented in this study are available on request from the corresponding author. The data are not publicly available due to [The project involves confidential issues since the experiments are carried out by different participants. Some of the parties wanted to keep the raw data confidential.].
